# IL-6-Producing, Noncatecholamines Secreting Pheochromocytoma Presenting as Fever of Unknown Origin

**DOI:** 10.1155/2016/3489046

**Published:** 2016-08-07

**Authors:** Marco Ciacciarelli, Davide Bellini, Andrea Laghi, Alessandro Polidoro, Antonio Pacelli, Anna Giulia Bottaccioli, Giuseppina Palmaccio, Federica Stefanelli, Piera Clemenzi, Luisa Carini, Luigi Iuliano, Cesare Alessandri

**Affiliations:** ^1^Department of Medico-Surgical Sciences and Biotechnologies, Internal Medicine Unit, ICOT Hospital, “Sapienza” University of Rome, Via Franco Faggiana 34, 04100 Latina, Italy; ^2^Department of Radiological Sciences, Oncology and Pathology, ICOT Hospital, “Sapienza” University of Rome, Via Franco Faggiana 34, 04100 Latina, Italy

## Abstract

Fever of unknown origin (FUO) can be an unusual first clinical manifestation of pheochromocytoma. Pheochromocytomas are tumors that may produce a variety of substances in addition to catecholamines. To date, several cases of IL-6-producing pheochromocytomas have been reported. This report describes a 45-year-old woman with pheochromocytoma who was admitted with FUO, normal blood pressure levels, microcytic and hypochromic anemia, thrombocytosis, hyperfibrinogenemia, hypoalbuminemia, and normal levels of urine and plasma metanephrines. After adrenalectomy, fever and all inflammatory findings disappeared.

## 1. Introduction

Petersdorf and Beeson [1] developed criteria for prolonged fevers, that is, fever of unknown origin (FUO), defined as fever ≥38.3°C (101°F) for >3 weeks that remains undiagnosed after a hospital workup. Being linked to more than 200 diseases, FUO remains one of the most difficult diagnostic challenges in medicine. Interestingly, FUO can be an unusual first clinical manifestation of pheochromocytoma [2]. In this report we describe a 45-year-old woman with pheochromocytoma who presented with FUO, normal blood pressure levels, normal levels of urine and plasma metanephrines, and laboratory findings suggestive of a marked inflammatory status.

## 2. Case

A 45-year-old woman was admitted to our Internal Medicine ward with FUO. She had been well until two months before presentation, when she had a left foot injury and few days later she began to have daily fever (continuous, without chills, and with fluctuations in body temperature between 38°C and 39°C). She saw her primary care physician who ordered an X-ray of left foot that did not show fractures. Because of persisting fever without associated symptoms, full blood count, inflammatory markers, urinary cultures, and chest X-ray were ordered. Laboratory tests revealed anemia (hemoglobin 10.2 g/dL, hematocrit 32.2%, and mean corpuscular volume 81 fl), a normal white cell and platelet count, and both an elevated erythrocyte sedimentation rate (ESR) (135 mm/h) and an elevated C-reactive protein (CRP) level (12 mg/dL, normal 0-1). Urinary cultures were negative and chest X-rays showed no infiltrates. Despite the absence of respiratory symptoms, she was treated empirically with a 10-day course of oral clarithromycin and i.m. ceftriaxone, without resolution of fever. After completion of antimicrobial therapy, she returned to her primary care physician who ordered further tests. Serologic tests for hepatitis B and hepatitis C viruses, cytomegalovirus (CMV), Epstein-Barr virus (EBV), and antistreptolysin O were negative. Tests for antinuclear antibodies (ANA), antineutrophil cytoplasmic antibodies (ANCA), and rheumatoid factor were also negative. A transthoracic echocardiogram was negative for vegetation. Since the fever persisted, the patient was referred to our hospital for further evaluation.

At the time of admission, she reported no associated chills, weight loss, night sweats, diaphoresis, anorexia, rash, cough, sputum production, chest or abdominal pain, palpitations, diarrhea, dysuria, headache, arthralgia, or myalgia. Her medical history was notable only for oophorectomy for a benign cyst 8 years earlier and hypothyroidism secondary to chronic lymphocytic thyroiditis. The only medication she was taking was levothyroxine 125 *μ*g. She had no known drug allergies. The patient lived in a small flat with her two healthy children and her husband, who was her only sexual partner, and there was no history of sexually transmitted disease or known exposure to tuberculosis infection. She worked as shop assistant in a lamp store. She had smoked 4-5 cigarettes daily for the past 4 years. She did not use illicit drugs and she drank one or two alcoholic beverages on social occasions. She reported no recent travel or contacts with sick persons. Her family medical history included ischemic heart disease and arterial hypertension in her parents.

At presentation, the patient's temperature was 39°C, heart rate 100 beats per minute, blood pressure 150/80 mmHg, respiratory rate 24 breaths per minute, and oxygen saturation 97% while she was breathing ambient air. She was alert and oriented and appeared well nourished and in no acute distress. There was no scleral icterus. The conjunctivae were slightly pale. Her oropharynx was clear and without erythema or exudates. The neck was supple, without palpable thyroid abnormalities. There were no rash or tick bites and no cervical, supraclavicular, axillary, or inguinal lymphadenopathy. The jugular venous pressure was 5 cm of water. Cardiac examination revealed a regular rhythm, normal S1 and S2 sounds, and a grade 2/6 holosystolic murmur at the apex radiating to the axilla. The lungs were clear on auscultation. The abdomen was soft, nontender, and nondistended, with no hepatosplenomegaly, masses, or abdominal bruits. Bowel sounds were normal. She had normal strength, sensation, gait, and coordination. There were no joint erythema or swelling and no peripheral edema.

Laboratory data on admission confirmed both an elevated ESR (127 mm/h) and an elevated CRP level (42.1 mg/dL). The white cell count was 7900/mm^3^, with 70.9% neutrophils, 19.5% lymphocytes, 8.9% monocytes, and 0.3% eosinophils. The hemoglobin level was 7.4 g/dL, the hematocrit 23.6%, the mean corpuscular volume 75 fL, and the platelet count 599000/*μ*L. The serum iron level was low (14 *μ*g/dL, normal 28–170) and ferritin level was normal (141 ng/mL, normal 10–295). The reticulocyte count was 1.4%. Thyroid function tests did not show hyperthyroidism (TSH 1.96 mIU/mL, normal 0.35–5.00; FT3 1.95 pg/mL, normal 2.00–3.90; FT4 1.05 ng/dL, normal 0.61–1.75). Fibrinogen level was markedly raised (1034 mg/dL, normal 170–410). Serum protein electrophoresis revealed both a markedly reduced albumin level (1.9 g/dL, normal 3.6–5.5) and raised alfa-1 (0.71 g/dL, normal 0.2–0.4) and alfa-2 level (1.71 g/dL, normal 0.5–1). The results of renal and liver function tests were normal, as were the results of coagulation tests and blood levels of electrolytes, glucose, creatine kinase, lactate dehydrogenase, and uric acid. Urinalysis was unremarkable, and tests for nitrites and leukocyte esterase were negative.

**Figure 1:**
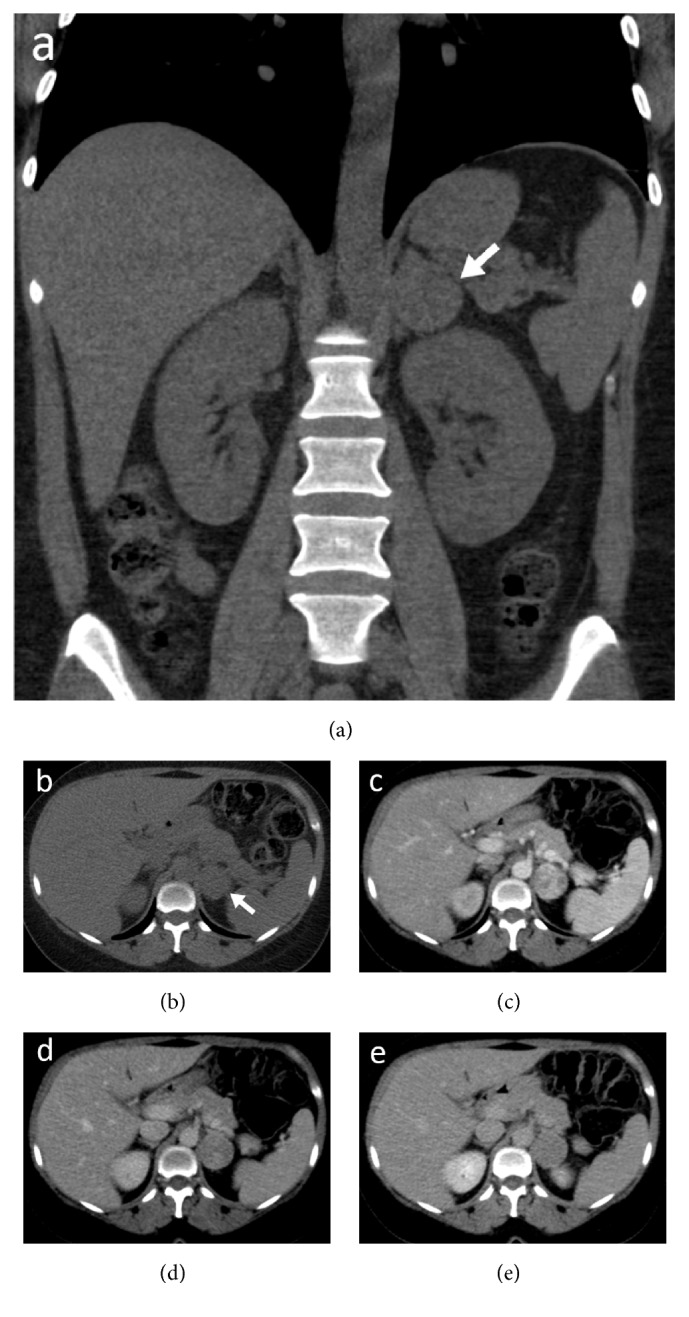
Coronal reformatted (a) and axial unenhanced CT images (b) show well-defined isodense mass in left adrenal gland measuring 3.5 cm in size. It is characterized by solid component (38 HU on unenhanced scan) (b), avid enhancement during arterial (c) and portal phases (102 HU) (d), and mild washout during delayed phase (79 HU) (e).

**Figure 2:**
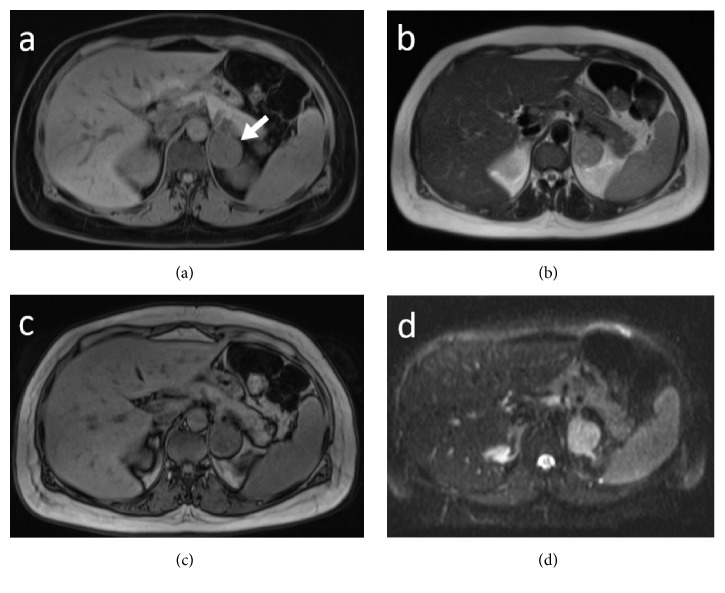
Axial T1-weighted (a) with fat suppression and T2-weighted (b) MR images show a left adrenal mass (arrow in (a)). The mass has heterogeneous high signal intensity on the T2-weighted image, low signal intensity on T1-weighted image, and no signal dropout on opposed phase T1-weighted image (c). Axial diffusion-weighted image (DWI) shows high signal intensity due to restricted diffusion (d).

Urinary cultures were negative. A blood culture was positive for* Rothia mucilaginosa* in one bottle of the first set. Three more sets of blood cultures revealed no bacterial growth. Since a transesophageal echocardiogram ruled out valvular vegetation, the blood culture positive for* Rothia mucilaginosa* was interpreted as a false positive result, probably due to contamination at the time of a difficult venipuncture. Test for antibodies to the human immunodeficiency virus (HIV) and Weil-Felix test were negative. Serum procalcitonin level was normal (0.29 ng/mL, normal 0.1–0.5). Tests for double-strand DNA antibodies were negative. C3 level was 2.78 mg/dL (normal 0.8–1.9) and C4 level was normal. Microcytic and hypochromic anemia and thrombocytosis were confirmed with peripheral blood smear. Esophagogastroduodenoscopy and colonoscopy did not show masses or sources of gastrointestinal bleeding. Thyroid and breast ultrasound showed no evidence of nodules or masses. CT of chest, abdomen, and pelvis showed an oval, well-defined, high attenuation mass on the left adrenal gland, characterized by intense and heterogeneous enhancement after i.v. administration of iodinated contrast media with mild washout (Figure 1). Hormonal tests revealed slightly increased morning serum fasting cortisol (28.5 *μ*g/dL, normal 5–25) and urinary cortisol (435 *μ*g/24 hours, normal 70–320) in the presence of normal ACTH (13 pg/mL, normal 1–46) and DHEA-S (58.4 *μ*g/dL, normal 35–430). Low-dose dexamethasone suppression test was performed and provided suppression of serum morning cortisol. Plasma aldosterone level and renin activity in the prone and upright positions were within the normal range. MRI with i.v. injection of paramagnetic-gadolinium-based contrast media was performed for further evaluation of the adrenal mass that showed low signal intensity on T1w image, high signal intensity on T2w image (light bulb sign), high signal intensity on diffusion-weighted images, demonstrating hypercellularity, and no loss of signal intensity on the out-of-phase T1w image, suggesting the absence of intracellular fat content (Figure 2). Given the high radiological suspicion of pheochromocytoma, further biochemical tests were performed. Urinary metanephrines levels were 863 *μ*g/L (normal 30–3000), plasma metanephrines levels were 48 pg/mL (normal 10–56), and chromogranin A levels were 95 ng/mL (normal 0–100). Bone marrow aspiration and biopsy showed no evidence of dysplasia or neoplasm. Whole-body 18F-FDG PET-CT confirmed a nodular lesion with markedly increased FDG uptake localized on left adrenal gland. Based on a previous report [2], we suspected that the clinical symptoms and abnormal laboratory findings were eventually dependent on IL-6 overproduction by the tumor. Actually IL-6 was found to be 180.28 pg/mL, while reported normal serum upper limits in healthy subjects range from 3 to 4 pg/mL [2, 3]. IL-1b, TNF-*α*, IFN-, IL-4, IL-5, IL-7, IL-8, IL-10, IL-12, IL-13, MCP-1, and MIP-1b either were undetectable or were within normal ranges. However, fever and marked inflammatory response were not suppressed by the administration of aspirin and doxazosin.

The patient was referred to another hospital to perform laparoscopic left adrenalectomy that provided almost immediate cessation of fever and normalization of the blood count and inflammatory markers levels. Histopathological analysis of the adrenal mass (diameter: 3 cm, weight: 53 g) showed chromaffin cells positive for synaptophysin and chromogranin A and negative for CAM 5.2. Surrounding sustentacular cells appeared to be positive for S-100 protein. Ki67 labeling index was <5% and there was no evidence of necrosis and vascular invasion. These findings were consistent with a pheochromocytoma.

## 3. Discussion

Pheochromocytoma is a tumor arising from chromaffin cells from the adrenal medulla. The most common manifestation of the disorder is sustained or paroxysmal hypertension due to excessive catecholamine release. It usually presents with typical signs and symptoms, such as headache, sweating, and palpitations. Pheochromocytomas are able to produce a variety of biologically active neuropeptides, hormones other than catecholamines, and cytokines, mainly IL-1 [4], IL-6, and TNF-*α* [5]. It has been suggested that interleukin-6 produced by the tumor gives rise to pyrexia in pheochromocytoma [2, 6, 7]. Our patient had high levels of IL-6 (180.28 pg/mL) but normal levels of IL-1 and TNF-*α*. There have been reports of adrenal tumors presenting with fever, hypertension, anemia, thrombocytosis, and hyperfibrinogenemia associated with elevated levels of IL-6 [3, 7]. As observed in previous cases of IL-6-producing pheochromocytoma [2, 8, 9] our patient had no hypertension but had fever, microcytic and hypochromic anemia, thrombocytosis, hyperfibrinogenemia, and hypoalbuminemia, suggestive of a marked inflammatory status. The absence of sustained or paroxysmal hypertension in our patient could be explained both by the normal catecholamines levels and by the increased nitric oxide synthesis due to IL-6 activity, which might have led to vasodilation [2, 9]. Although the effectiveness of nonsteroidal anti-inflammatory drugs [8, 10] and alpha-adrenergic receptor blockers [2, 6] in reducing inflammatory symptoms has been shown in some patients with IL-6-producing pheochromocytoma, our patient did not achieve fever remission through the use of both aspirin and doxazosin.

Our patient had slightly increased morning serum fasting cortisol (28.5 *μ*g/dL, normal 5–25) and urinary cortisol (435 *μ*g/24 hours, normal 70–320), as previously observed in a patient affected by IL-6-producing pheochromocytoma [9]. Interestingly, it has been shown that exposure to IL-6 can increase cortisol release from human, bovine, and rat adrenal cells [11]. Furthermore, several cases of Cushing's syndrome due to ACTH-secreting pheochromocytoma have been reported [12, 13]. However, our patient had normal plasma ACTH level (13 pg/mL, normal 1–46), physical examination revealed no physical signs of cortisol overproduction, and the low-dose dexamethasone suppression test showed suppression of serum morning cortisol.

Although FUO has been observed among both children [14] and adults [15] with pheochromocytoma, to the best of our knowledge this is the first case reporting a patient with IL-6-producing, noncatecholamines secreting, pheochromocytoma presenting as FUO.

In accord with previous case reports, pheochromocytoma must be considered in the differential diagnosis of FUO, and the assay of serum IL-6 levels emerges as a precious marker in the laboratory workup. In addition, IL-6 has relevance in clinical management because resection of IL-6-secreting pheochromocytoma is followed by complete resolution of both fever and other inflammation-associated signs.
